# Digital rehabilitation in a low-resource setting: lessons from building an integrated ecosystem in Burundi

**DOI:** 10.3389/fdgth.2026.1813216

**Published:** 2026-04-10

**Authors:** Jean Mapinduzi, Olivier Jadin, Gérard Ndacayisaba, Joachim Van Cant, Bruno Bonnechère

**Affiliations:** 1 TechnoRehab Lab2, Filière de Kinésithérapie et de Réadaptation, Département des Sciences Cliniques, Institut National de Santé Publique (INSP), Bujumbura, Burundi; 2 REVAL Rehabilitation Research Center, Faculty of Rehabilitation Sciences, Hasselt University, Diepenbeek, Belgium; 3 Technology-Supported and Data-Driven Rehabilitation, Data Sciences Institute, Hasselt University, Diepenbeek, Belgium; 4 APEFE, Physical Medicine and Rehabilitation Program, Office APEFE Bujumbura, Bujumbura, Burundi; 5 CNRKR - Centre National de Référence en Kinésithérapie et Réadaptation Médicale, Bujumbura, Burundi; 6 Unité de Recherche en Sciences de la Réadaptation/Rehab Lab, Faculté des Sciences de la Motricité Humaine, Université Libre de Bruxelles, Bruxelles, Belgique; 7 Department of PXL – Healthcare, PXL University of Applied Sciences and Arts, Hasselt, Belgium

**Keywords:** access to care, capacity building, digital rehabilitation, health policy and systems research, health systems strengthening, integrated care, rehabilitation

## 1 The rehabilitation gaps

Rehabilitation services encompass a range of interventions designed to enhance and restore functional ability and quality of life to those with physical impairments, cognitive limitations, or psychological conditions (1). These services are crucial for individuals recovering from injuries, managing chronic illnesses, or coping with disabilities, promoting independence and social inclusion.

Globally, over 2.4 billion people live with conditions that require rehabilitation services (2). Most of these individuals reside in low- and middle-income countries (LMICs), where access to effective rehabilitation is often severely limited. This gap is particularly acute in countries emerging from conflict, experiencing humanitarian crises, or facing high burdens of chronic diseases and disability (3).

Burundi, one of the world's poorest nations with a population of approximately 14.3 million (4), faces significant healthcare challenges. The country has experienced decades of political instability and civil conflict, which have severely impacted its health infrastructure. Burundi faces a substantial burden from both communicable and non-communicable diseases. While progress has been made in reducing child mortality and improving vaccination rates, the country still grapples with high rates of diseases like malaria, HIV/AIDS, and various neglected tropical diseases. Since a few years, non-communicable diseases (NCDs) are on the rise, contributing significantly to the overall disease burden, making up 43% of deaths (5). Rehabilitation services have historically been neglected, with minimal investment in human resources, facilities, and equipment (6), it is estimated than less than 10% of the patients requiring rehabilitation in Burundi can access rehabilitation services (7, 8). The burden of disability in Burundi is substantial, exacerbated by conflict-related injuries, infectious diseases, non-communicable conditions, and limited access to maternal and child health services (9).

However, over the last years, a notable exception has emerged through the establishment and growth of a comprehensive rehabilitation ecosystem in Bujumbura. This model was developed through a development project initiated in 2011 by the Belgian organization APEFE, which developed the same strategic approach in Burkina Faso and Benin. Funding was provided by the Belgian government. Several Belgian universities [UCLouvain, UHasselt and Université Libre de Bruxelles (ULB)] were closely involved in the implementation of the activities. This opinion explores this model, arguing that its success stems from a deliberate and integrated approach to education, research, and clinical care, and its grounding in digital health policy and systems that should be implemented in other LMICs. As an opinion article, this paper presents a viewpoint based on the authors’ collective implementation experience and published evidence, rather than reporting new primary research data.

## 2 The integrated rehabilitation ecosystem: a tripartite approach

**Figure 1:**
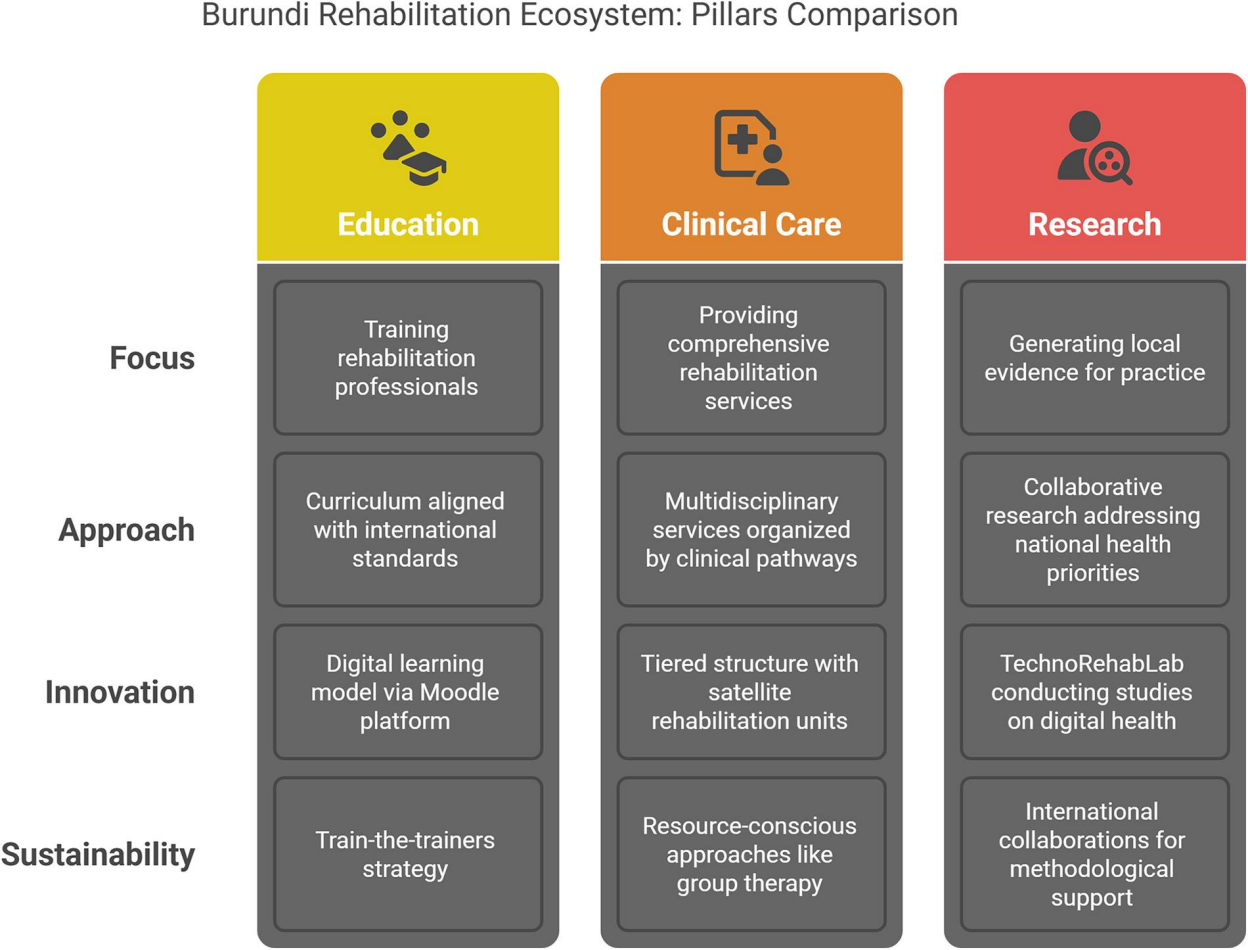
Description of the three pillars related to the development of (digital) rehabilitation in Burundi.

The rehabilitation ecosystem established in Burundi, and in particular at the National Institute of Public Health (INSP), represents a comprehensive approach to addressing the above-mentioned challenges. The ecosystem is built upon three interconnected pillars: education, clinical care, and research; each of these components integrates a strong digital component to further strengthen and speed up development, implementation and sustainability (10). This tripartite structure enables synergistic interactions that strengthen each component while contributing to the overall sustainability and effectiveness of healthcare and rehabilitation services in Burundi (Figure 1).

### 2.1 Education: building a sustainable workforce

The School of Physiotherapy and rehabilitation serves as the cornerstone of the rehabilitation ecosystem. Established in 2019 with a commitment to developing locally-trained professionals, the school offers a three-year bachelor's degree program in physiotherapy and rehabilitation. The curriculum aligns with international standards while being adapted to local health priorities. It combines theoretical foundations with extensive practical skills and emphasizes conditions highly prevalent in the Burundi context, including musculoskeletal disorders, neurological conditions, paediatric disabilities, cardiopulmonary conditions, and trauma rehabilitation.

Each year, approximately twenty students graduate from the program. Given the persistent national shortage of rehabilitation professionals, nearly all graduates are rapidly integrated into the workforce, though retention remains a challenge, with 20 to 25% seeking opportunities abroad. To address this risk, over time, the school has gradually expanded its academic staff by incorporating local physiotherapists who received advanced training abroad – primarily at University of Abomey-Calavi (Benin) for bachelor's degrees and at UCLouvain for master's and doctorate degrees. This “train-the-trainers” strategy of has been crucial for sustainability of rehabilitation program, reducing dependence on international expertise while building local academic leadership.

Additionally, the school has established partnerships with regional and international institutions, including the Institut Supérieur des techniques Médicales de Bukavu (DRC), UHasselt, UCLouvain, and ULB, facilitating knowledge exchange and academic development.

One of the school's flagship pedagogical initiatives is a project titled “Implementation of a device to help learn clinical reasoning in physiotherapy as part of the bachelor's program in Physiotherapy and Rehabilitation of the INSP in Bujumbura”. This ambitious initiative responds to an internal curriculum reform and is based on international standards including World Physiotherapy and WHO recommendations (11). This project aims to equip physiotherapy students with advanced competencies in clinical reasoning by focusing on essential skills such as patient evaluation, diagnosis, planning, and therapeutic decision-making in various clinical contexts through an integrated and longitudinal approach.

To ensure robust knowledge acquisition, and foster self-directed learning, a comprehensive digital learning model has been implemented via the Moodle platform. Students engage in diverse learning activities, including quizzes and contextualized clinical cases resolution. Special emphasis is placed on interactive clinical vignettes tailored to the Burundian context, which simulate real-life decision-making scenarios. These are complemented by metacognitive exercises, problem-based learning modules, and script concordance tests, collectively encouraging reflective and adaptive reasoning. Tools such as Wooclap are also used in real time to enhance classroom engagement and stimulate critical thinking.

Beyond digital strategies, the project also integrates real-world experiential strategies. Students participate in simulated patient encounters and objective structured clinical examinations to assess their clinical reasoning under standardized and realistic conditions. In parallel, they engage in supervised clinical activities with real patients during practical sessions, reinforcing their ability to apply theoretical knowledge in authentic situations.

Throughout their training, students are required to build digital and paper-based clinical portfolios, based on real patient encountered during internships. These portfolios include critical reflection, evidence-informed analysis, and case documentation, serving as key tools for learning and assessment, particularly during the final synthesis exam and oral defence.

This multifaceted pedagogical strategy—anchored in contextual relevance, evidence-based methods, and technological innovation—seeks not only to improve clinical reasoning competencies, but also to support the professional identity development of future Burundian physiotherapists.

The educational effort also extends beyond pre-service training. Ongoing professional development for practicing physiotherapists and other healthcare workers is supported through regular workshops and short courses, allowing continuous integration of new knowledge and techniques into the national health system.

The educational transformation is the result of a strong partnership between the ULB and the INSP in Bujumbura, with financial support of ARES-CCD (Académie de Recherche et d’Enseignement Supérieur – Commission de la Coopération au Développement).

### 2.2 Clinical care: providing model services

The second pillar of the ecosystem is clinical care, centered around a model rehabilitation center at the INSP. This center provides comprehensive rehabilitation services while serving as a clinical training site for students and, later, a platform for implementing and evaluating evidence-based practices.

The rehabilitation center employs a multidisciplinary approach. Services are organized around key clinical pathways for common conditions, ensuring standardized, high-quality care (12). The center has gradually expanded its technological capacity, incorporating essential equipment for assessment and treatment while maintaining a focus on interventions that are sustainable in the Burundian context.

A distinctive feature of the clinical care component is its tiered structure, with the central facility linked to satellite rehabilitation units in provincial hospitals (*n* *=* 44). This network approach extends the reach of rehabilitation services while providing varied clinical experiences for students and opportunities for decentralized research. In 2023, 32,995 patients received treatment across the network of centers, totaling 164,675 sessions. By 2024, these figures rose significantly to 43,289 patients and 216,445 sessions—marking a 31% increase in both patient volume and session count. This growth reflects expanding demand for services and the commitment to scaling healthcare access. By the end of 2024, 148 physiotherapists were registered in the country.

The clinical services incorporate innovative approaches to address resource constraints, including group therapy sessions, peer support networks, and caregiver training programs. Digital health technologies, including mobile applications for home exercise programs (13), teleconsultation for remote areas (14) and serious games (15) are being piloted to extend the reach of limited human resources.

### 2.3 Research: generating contextually relevant evidence around digital health

The research pillar of the ecosystem addresses the critical need for locally-relevant evidence to guide rehabilitation practice and policy in Burundi. The INSP has established a *TechnoRehabLab* (16), a unique rehabilitation research unit that conducts studies on rehabilitation needs, intervention effectiveness, and service delivery models appropriate for the Burundian context. The development of this centre is based on collaboration between the INSP and the UHasselt and is based upon the development of other *TechnoRehabLab* in Belgium and Africa, and it was supported by the VLIR-UOS.

Research activities are strategically aligned with national health priorities and digitalization and focus on pragmatic questions that can directly inform practice (17). Key research themes include mHealth (13), remote monitoring (18), telerehabilitation (19), serious games (20) and virtual reality (21). Research focus on the acceptability of these new technologies by both the patients and clinicians, feasibility and effectivity studies.

The research unit employs a collaborative approach, engaging clinicians, students, patients, and community members in the research process. This participatory methodology enhances the relevance and applicability of findings while building research capacity across the ecosystem. International collaborations provide methodological support and opportunities for Burundian researchers to contribute to global knowledge in rehabilitation. Since its opening in 2024, 3 research papers about digital rehabilitation have been published in international peer-reviewed journal (20, 22, 23), and 3 studies are currently being performed on the acceptability and efficacy of mHealth and serious games.

## 3 Integration and coordination: the key to a successful rehabilitation ecosystem

The success of the rehabilitation ecosystem in Burundi can be attributed not only to the strength of its individual components but also to its strategic integration within the broader health system and a clear ambition to fully integrate digitalization within the healthcare system. This integration has been guided by health policy and systems research (HPSR) principles (24), ensuring alignment with national priorities and sustainability within existing structures (25).

At the governance level, the Institute has worked closely with the Ministry of Health to develop national rehabilitation policies and standards. This collaboration has resulted in the inclusion of rehabilitation in essential health service packages and national health insurance schemes, addressing critical financing barriers. The ecosystem's leaders have advocated for rehabilitation to be recognized as an integral component of universal health coverage, rather than a luxury service.

Human resource strategies have focused on official recognition of rehabilitation professions within the national health workforce, with standardized job descriptions, career pathways, and appropriate remuneration. This formalization has been crucial for retention of graduates within the public health system.

Information systems have been strengthened to include rehabilitation indicators in national health information frameworks, enabling monitoring of service coverage and outcomes. This data-driven approach has been valuable for demonstrating the impact of rehabilitation services and advocating for continued investment.

## 4 Health policy and system research: informing the development of the rehabilitation ecosystem

The development of the rehabilitation ecosystem in Burundi has been informed by HPSR (24), which has played a critical role in shaping the country's healthcare system and rehabilitation services. HPSR has enabled policymakers and stakeholders to identify priority areas for investment, develop context-specific solutions, and evaluate the effectiveness of rehabilitation programs (26).

The integration of HPSR into the rehabilitation ecosystem in Burundi has several benefits. Firstly, it enables the development of evidence-based policies and programs that address the needs of people with disabilities and chronic conditions. Secondly, it facilitates the evaluation of existing rehabilitation services, identifying areas for improvement and optimizing resource allocation. Finally, it ensures that rehabilitation services are aligned with the broader healthcare system, promoting coordination and integration of care.

## 5 Challenges, opportunities, and scalability

Despite the successes, the INSP rehabilitation ecosystem faces ongoing challenges. These challenges require innovative solutions and sustained political commitment; a key summary of action points is presented in Table 1. A key challenge is the translation from research to clinics. While promising results were obtained using VR for neurorehabilitation, initial attempts to implement VR therapy failed due to equipment costs; lessons informed the shift to mHealth solutions and prioritize low-tech solutions for rural service delivery. Another important challenge is the important reduction of international funding for cooperation undermining the possibilities for future collaboration, mainly in terms of research and development of new centres, with the teaching and operation of clinical centres being taken over by the government.

**Table 1:** Eleven key challenges facing the rehabilitation ecosystem in Burundi, along with associated stakeholders, risks, and mitigation strategies.

Challenge^a^	Description	Key involved partners^b^	Risk associated	Severity^c^	Mitigation strategies^d^
Funding Sustainability	Reliance on external grants and donor funding creates instability. Core operational costs are often not fully covered.	INSP, Ministry of Health (MoH), Donors (e.g., APEFE & Belgian Government, World Bank, foundations), Partner NGOs	• Program disruption • Reduced service delivery • Inability to maintain infrastructure & equipment	8/10 Program disruption very likely without diversified funding	• Diversify funding sources (government allocation, private sector partnerships, fee-for-service options where appropriate) • Develop a long-term financial plan, build a strong fundraising team, advocate for increased government investment
Human Resource Capacity – Retention & Brain Drain	Difficulty attracting and retaining qualified rehabilitation professionals (physiotherapists, technicians) due to limited career opportunities, low salaries, and better prospects elsewhere.	INSP, Universities (training institutions), MoH, Government (civil service policies)	• Threats to the training capacities of physiotherapists at the INSP • Reduced quality of care • Service gaps • Slowed expansion of rehabilitation services • Instability of rehabilitation services	9/10 Threatens training and service continuity	• Enhance training programs • Improve salary scales (advocate with MoH) • Create professional development opportunities • Build a supportive work environment • Establish mentorship programs • Offer scholarships/loan repayment assistance
Limited Infrastructure & Equipment	Inadequate infrastructure (clinic space, rehabilitation equipment, lack of internet connection and power supply) hindering the provision of comprehensive services.	INSP, MoH, Donors, Local construction companies	• Reduced service capacity • Inability to treat complex cases • Lower quality of care	7/10 Caps service expansion	• Phased infrastructure development plan • Prioritize essential equipment procurement • Explore cost-effective equipment options (e.g., locally made assistive devices) • Seek donations of equipment • Ensure proper maintenance and repair of existing equipment
Assistive Device Access & Affordability	High cost and limited availability of assistive devices (wheelchairs, prosthetics, orthotics) create barriers to access for people with disabilities. Lack of a training school in orthopaedic technology/ Orthopaedic equipment Lack of local qualified professionals in orthopaedic technology	INSP, MoH, Local manufacturers/importers, NGOs specializing in assistive technology, Users and their families	• Limited functional independence • Reduced participation in society • Increased dependence on caregivers • Disruption of services in assistive technologies upon the retirement of existing qualified professionals	8/10 Restricted autonomy and functional independence	• Develop a national assistive device policy • Explore local manufacturing options • Negotiate bulk purchasing agreements • Establish a loan/subsidy program for assistive devices • Promote community-based rehabilitation approaches (utilizing locally available materials) • Search for scholarship for young professional in orthopaedic technology Develop/create a training school in orthopaedic technology
Community Awareness & Stigma	Low public awareness about rehabilitation services and persistent stigma associated with disability, leading to delayed seeking of care and social exclusion.	INSP, MoH, Community leaders, Local media, NGOs working with disability rights	• Low utilization of services • Social isolation • Limited opportunities for people with disabilities	6/10 Delays care-seeking	• Public awareness campaigns (radio, television, community events) • Advocacy for inclusive policies • Training for healthcare workers on disability awareness • Support for self-help groups and disability rights organizations
Integration with Primary Healthcare	Lack of integration of rehabilitation services into primary healthcare facilities, resulting in limited access for people in rural areas.	INSP, MoH, Primary Healthcare Facilities, District Health Teams	• Unequal access to care • Increased burden on specialist centers • Delayed rehabilitation interventions	7/10 Urban-rural disparity	• Train primary healthcare workers in basic rehabilitation techniques • Develop referral pathways between primary and specialist care • Integrate rehabilitation screening into routine healthcare visits • Establish mobile rehabilitation clinics
Data Collection & Monitoring	Weak data collection systems hinder effective monitoring and evaluation of rehabilitation programs, making it difficult to demonstrate impact and allocate resources effectively.	INSP, MoH, Health Information Systems Department	• Inaccurate program evaluation • Difficulty demonstrating impact Inefficient resource allocation	5/10 Hinders impact evidence	• Strengthen data collection tools and protocols • Integrate rehabilitation data into the national health information system • Train healthcare workers on data collection procedures • Use data to inform program planning and decision-making • Develop contextually-adapted data collection tools like questionnaires
Policy & Regulatory Framework	Lack of a comprehensive national policy and regulatory framework for rehabilitation, creating uncertainty and hindering long-term planning.	INSP, MoH, Ministry of Social Affairs, Disability, Rights Organizations, Legal experts	• Inconsistent service delivery • Lack of accountability • Limited protection of the rights of people with disabilities	6/10 Fragmented services	• Advocate for the development and implementation of a national rehabilitation policy • Establish clear standards for rehabilitation practice • Strengthen legal protections for people with disabilities
Political Instability & Conflict	Ongoing political instability and potential for conflict such as the war in the DR Congo, only a few kilometres from Burundi, can disrupt rehabilitation services and exacerbate existing challenges.	INSP, MoH, Government, International humanitarian organizations	• Disrupted service delivery • Displacement of patients and healthcare workers • Increased demand for rehabilitation services	9/10 Unpredictable disruptions	• Develop a contingency plan for emergencies, establish partnerships with humanitarian organizations • Advocate for the protection of healthcare facilities and personnel during conflict
Coordination among Stakeholders	Poor coordination among different stakeholders involved in rehabilitation (government agencies, NGOs, international organizations) leading to duplication of efforts and inefficient use of resources.	INSP, MoH, NGOs, Donors, Disability Rights Organizations	• Fragmented services • Wasted resources • Lack of synergy	5/10 Suboptimal resource allocation	• Establish a national rehabilitation coordination committee • Develop a shared strategic plan • Promote information sharing and collaboration among stakeholders
Poor economic access to rehabilitation care	Despite the strengthening of healthcare provision, the lack of financial support mechanisms limits the economic access of the poorest to rehabilitation care.	MoH, Government international organizations such as EU, World Bank, … to help building Universal Health Coverage	• A big (major) part of the population does not have access to rehabilitation services	8/10 Excludes poorest	• Make the Burundian government work in agreement with major international funding agencies to build a viable UHC system

^a^ Challenges identified in this table represent a synthesis of the authors’ collective implementation experience over more than a decade of developing and managing rehabilitation services in Burundi. these challenges are presented as expert perspectives rather than findings from formal qualitative research.

^b^ Partners listed are identified based on the authors’ direct experience with stakeholder engagement in the Burundian rehabilitation ecosystem, including formal partnerships, collaborative agreements, and ongoing working relationships. Their inclusion reflects their actual roles in service delivery, policy development, training, research, or funding, rather than findings from a systematic stakeholder analysis.

^c^ Severity ratings (scale 1–10) represent the authors’ expert consensus based on collective assessment of each challenge's potential impact on rehabilitation service delivery and sustainability in Burundi. Ratings were developed through discussion among the author team (which includes individuals with clinical, academic, and policy expertise) and consider each challenge's capacity to disrupt services, affect patient outcomes, or undermine ecosystem sustainability. These are qualitative judgments, not quantitative measurements.

^d^ Mitigation strategies are proposed based on: (i) approaches partially implemented and showing promise in the Burundian context; (ii) recommendations from the global rehabilitation literature (as referenced throughout the manuscript); (iii) WHO guidelines and frameworks (e.g., Rehabilitation 2030, Global Strategy on Digital Health); and (iv) lessons learned from challenges encountered during implementation. These are offered as perspectives for discussion and contextual adaptation, not as proven interventions requiring validation through formal research.

However, these challenges also present opportunities. The INSP model can serve as a demonstration site for scaling up rehabilitation services nationally and within the region using digital solutions. While this model reflects the specific Burundian context, its core principles—strategic integration of education, clinical care, and research—may inform rehabilitation strengthening efforts in other LMICs, with necessary contextual adaptations. Building on the WHO Global Strategy on Digital Health (27) and Rehabilitation 2030 (28), our model presents a structured pathway for scaling rehabilitation services in similar LMIC contexts. We propose a four-step replication strategy emphasizing flexibility and sustainability: (i) Contextual needs assessments to align services with local disease burden and infrastructure capacity; (ii) Early policy integration through coordinated engagement with government stakeholders and development partners; (iii) Phased geographic rollout, initially prioritizing urban centers as training hubs before expanding periphery services; and (iv) Strategic digital adaptation using WHO-recommended, low-bandwidth tele-rehabilitation tools to overcome connectivity barriers. This tiered approach mirrors WHO's digital rehabilitation pillars—service delivery, workforce, governance, and technology—while incorporating lessons from our implementation challenges regarding cost-intensive modalities. The framework maintains particular emphasis on political-economic realities through its donor coordination mechanisms and graduated service expansion, offering a pragmatic balance between evidence-based standards and contextual feasibility.

The INSP's integrated model provides a pathway toward achieving Universal Health Coverage in Burundi and beyond, recognizing rehabilitation as an essential health service that contributes to improved health outcomes, reduced disability, and enhanced quality of life and productivity.

## 6 Conclusion and perspectives

The integrated rehabilitation ecosystem developed in Burundi illustrates that comprehensive rehabilitation services can be successfully established in resource-limited settings through strategic integration of education, research, and clinical care within the health system framework. This model offers perspectives that may inform other low-income countries seeking to develop sustainable rehabilitation services, recognizing that each context requires its own adapted approach.

By investing local capacity-building, generating contextually relevant evidence, and providing model clinical services, the INSP of Bujumbura has created a foundation for addressing the rehabilitation needs of Burundi's population. The continued evolution of this ecosystem will contribute not only to improved health outcomes but also to broader social inclusion and economic participation of persons with disabilities in Burundi.

To strengthen the model's scalability and replicability, future efforts should focus on systematically collecting and reporting impact data, such as employment rates of graduates, regional coverage of rehabilitation services, user satisfaction, and scientific output. In parallel, developing a visual framework that clearly illustrates the model's tripartite logic and digital components would enhance its dissemination and uptake.

Furthermore, as digital health tools become central to the ecosystem, addressing the digital divide remains crucial. Ensuring equitable access, particularly in rural areas and among professionals with limited digital literacy, will be essential to maximize the benefits of these innovations without reinforcing existing disparities. Several solutions are currently being investigated to mitigate this risk such as a hybrid training for community health workers in paper-based alternatives where internet is unreliable or try to develop partnerships with telecom providers to subsidize data costs for tele rehab users.

Looking ahead, the sustained evolution of this ecosystem depends on continued investment in local leadership, strong academic and clinical partnerships, and a shared commitment to inclusive health systems strengthening. The Burundian experience may thus serve as a source of inspiration and learning for rehabilitation development in similar contexts worldwide, encouraging constructive discussion and adaptation rather than direct replication. While this model reflects the specific Burundian context, its core principles - strategic integration of education, clinical care, and research - may inform rehabilitation strengthening efforts in other LMICs, with necessary contextual adaptations
